# Glenohumeral interposition of rotator cuff stumps: a rare complication of traumatic rotator cuff tear*

**DOI:** 10.1590/0100-3984.2013.0011

**Published:** 2016

**Authors:** Paulo Moraes Agnollitto, Marcio Wen King Chu, Mario Muller Lorenzato, Salomão Chade Assan Zatiti, Marcello Henrique Nogueira-Barbosa

**Affiliations:** 1 Physician Assistant at Division of Radiology, Hospital das Clínicas - Faculdade de Medicina de Ribeirão Preto da Universidade de São Paulo (HCFMRP-USP), Ribeirão Preto, SP, Brazil.; 2 Volunteer Physician, Fellow of Musculoskeletal Radiology, Division of Radiology at Hospital das Clínicas - Faculdade de Medicina de Ribeirão Preto da Universidade de São Paulo (HCFMRP-USP), Ribeirão Preto, SP, Brazil.; 3 MD, Radiologist, Clínica Radiologia Especializada, Ribeirão Preto, SP, Brazil.; 4 MD, Orthopedist, Hospital Especializado de Ribeirão Preto, Ribeirão Preto, SP, Brazil.; 5 Associate Professor of Radiology, Centro de Ciências das Imagens e Física Médica (CCIFM) - Faculdade de Medicina de Ribeirão Preto da Universidade de São Paulo (FMRP-USP), Ribeirão Preto, SP, Brazil.

**Keywords:** Interposition, Rotator cuff, Tear, Trauma, Shoulder, Magnetic resonance imaging

## Abstract

The present report describes a case where typical findings of traumatic glenohumeral interposition of rotator cuff stumps were surgically confirmed. This condition is a rare complication of shoulder trauma. Generally, it occurs in high-energy trauma, frequently in association with glenohumeral joint dislocation. Radiography demonstrated increased joint space, internal rotation of the humerus and coracoid process fracture. In addition to the mentioned findings, magnetic resonance imaging showed massive rotator cuff tear with interposition of the supraspinatus, infraspinatus and subscapularis stumps within the glenohumeral joint. Surgical treatment was performed confirming the injury and the rotator cuff stumps interposition. It is important that radiologists and orthopedic surgeons become familiar with this entity which, because of its rarity, might be neglected in cases of shoulder trauma.

## Abstract (pt)

O objetivo deste trabalho é relatar um caso com os achados de imagem característicos da lesão traumática do manguito rotador com interposição de múltiplos cotos de tendões do manguito rotador entre a glenoide e o úmero, confirmada cirurgicamente. Esta condição é uma rara complicação do trauma do ombro. Em geral ocorre no trauma de alta energia, frequentemente associada com luxação da articulação glenoumeral. No caso em questão não houve documentação de luxação franca da articulação glenoumeral. As radiografias mostraram aumento do espaço articular, rotação interna do úmero e fratura do coracoide. Nas imagens de ressonância magnética, além das alterações observadas nas radiografias, foi identificada ruptura massiva do manguito rotador com interposição dos cotos dos tendões do supraespinal, do infraespinal e do subescapular no interior da articulação glenoumeral. Foi realizado tratamento cirúrgico confirmando a lesão e a interposição dos cotos do manguito. É importante que radiologistas e ortopedistas estejam familiarizados com esta entidade, que, pela sua raridade, pode ser negligenciada no atendimento do trauma do ombro.

## INTRODUCTION

Traumatic rotator cuff injury is rarely observed in young patients and generally occurs after high-energy trauma. Even more rarely, traumatic rotator cuff injury may be associated with interposition of stumps or more than one of the rotator cuff tendons between the humerus and the glenoid^(1-5)^. In the case of rotator cuff stumps interposition in the glenohumeral joint (GHJ), the most common clinical presentation is articular blockage, many times with persistence of subluxation and/or irreducible luxation of the GHJ^(4-8)^.

The objective of this report is to describe a surgically confirmed case with characteristic imaging findings of traumatic rotator cuff injury with multiple stumps interposition between the glenoid and the humerus.

## CASE REPORT

A female, 27-year-old patient was referred to the emergency unit, at another service, with a history of motorcycle accident occurred about two hours before her arrival at the unit. She presented with pain and limited range of motion in her right shoulder. At the initial approach, there was no report of GHJ dislocation, and the patient was discharged with analgesic medication. No supplementary tests or imaging studies were performed.

**Figure 1:**
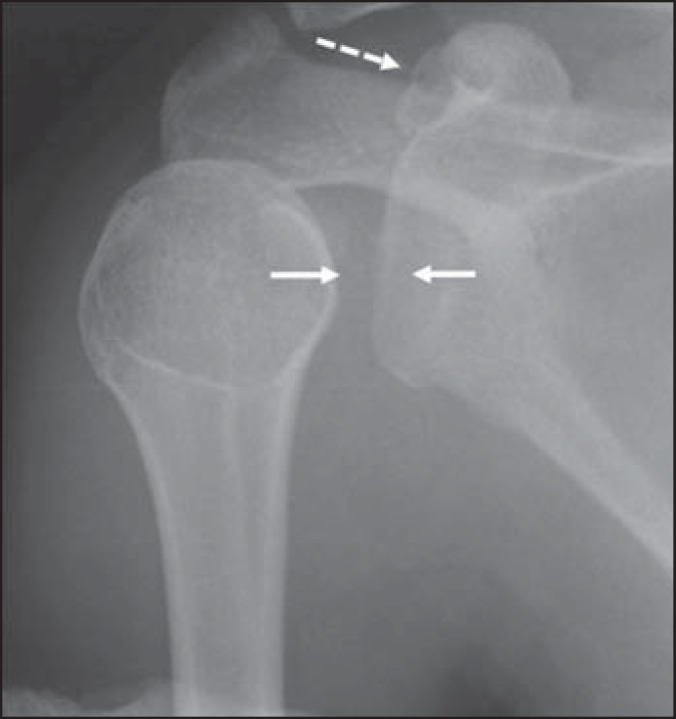
Radiography of right shoulder. Anteroposterior view showing glenohumeral joint space widening (continuous arrows). The humerus presented with internal rotation. The dashed arrow indicates fracture of the coracoid process.

The patient evolved with right shoulder pain and block-age and, 15 days after the episode, she sought specialized assistance, undergoing plain radiography of her right shoulder. The analysis of the images showed coracoid process fracture, GHJ space widening, and internal humeral rotation (Figure 1). No signs of glenohumeral dislocation or instability were found at the radiographic images.

**Figure 2:**
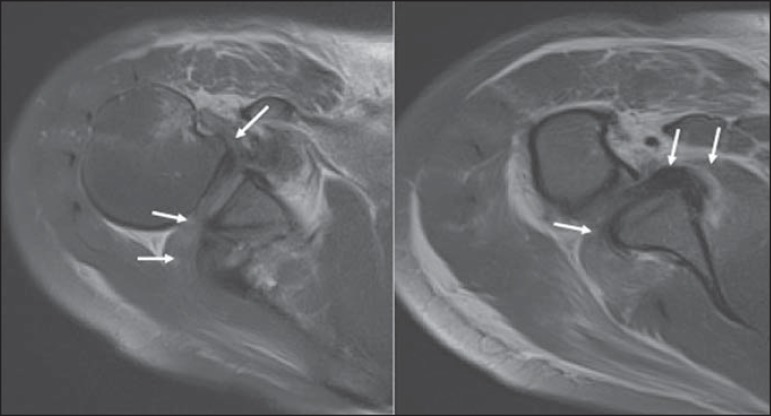
MRI. Axial sections images acquired with intermediate weighting demonstrates rotator cuff stumps interposition (arrows) between the glenoid and the humerus, explaining the blockage and the joint space widening.

**Figure 3:**
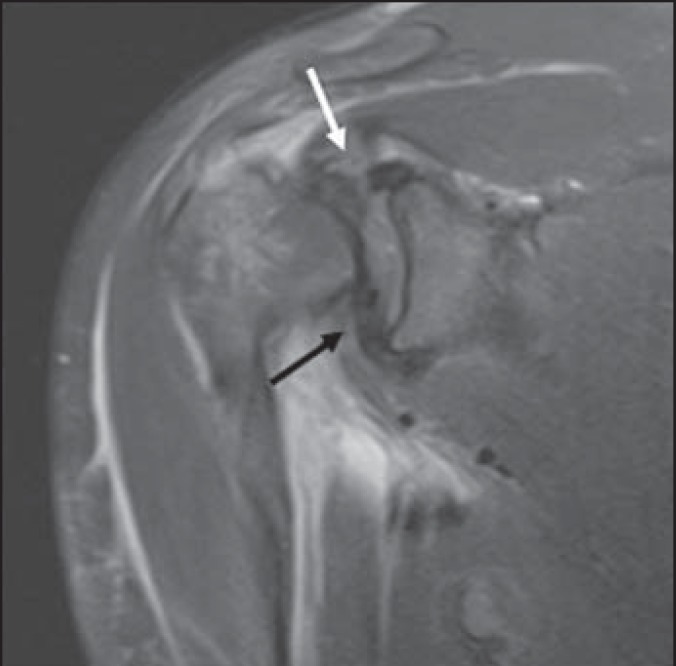
MRI, coronal section, intermediate weighting. The arrows identify rotator cuff stumps interposition between the glenoid and the humerus. The white arrow indicates the supraspinatus tendon and the black arrow shows the subscapularis tendon.

The hypothesis of traumatic rotator cuff tear was raised on the basis of the plain radiography findings, and the patient was submitted to magnetic resonance imaging (MRI) whose images demonstrated the presence of a traumatic injury with interposition of the supraspinatus, infraspinatus and subscapularis tendons stumps in the GHJ (Figures 2 and 3). Coracoid process fracture, diffuse periarticular edema, and edema of intermuscular fat planes were also identified.

**Figure 4:**
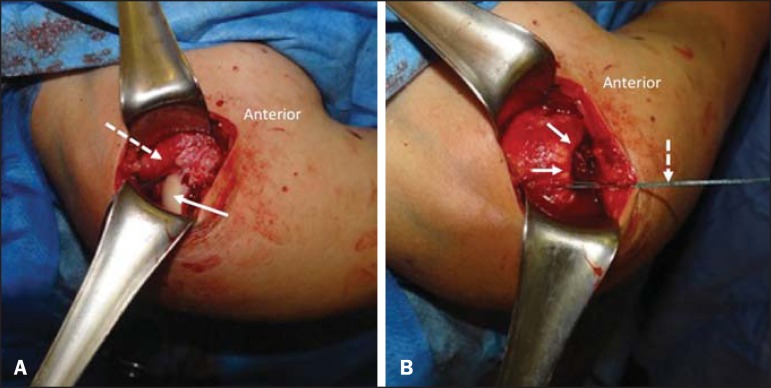
**A:** Intraoperative finding demonstrating uncovered humeral head (continuous arrow) and retracted and interposed rotator cuff stumps in the glenohumeral joint (dashed arrow). **B:** Intraoperative finding demonstrating rotator cuff (continuous arrows), repaired by surgical thread (dashed arrow) coursing up to the site of insertion of the greater humeral tuberosity.

Then, the patient was submitted to open surgical exploration, with diagnostic confirmation and rotator cuff reinsertion (Figure 4).

The patient presented a good evolution over the immediate postoperative period and was discharged. Three months after the surgery, she presented with external rotation restriction and was submitted to arthroscopy that revealed the presence of adhesions, which were released. After this new peocedure, the patient evolved satisfactorily, without any new complication.

## DISCUSSION

Traumatic rotator cuff stumps interposition in the GHJ is a very rare complication in shoulder trauma. In general, it occurs as a result from high-energy trauma, and frequently is associated with either anterior or posterior GHJ luxation. Relatively few articles on this subject are found in the literature, and most of them are case reports.

Difficulty or incapacity to reduce GHJ luxation is not common^(1-3)^. Irreducible GHJ luxation may be related to bone tissue or soft tissues interposition^(1,2,4,6-10)^. Amongst the causes associated with soft tissues interposition, one can mention, for example, interposition of the long head of biceps^(9)^ and interposition of the musculocutaneous nerve^(10)^; however, interposition of rotator cuff tendons, particularly the subscapularis tendon, is highlighted^(2,4,5,7,8)^. Soft tissues interposition and bone tissue interposition may occur concomitantly^(2)^.

Most cases reported in the literature are associated with high-energy trauma, with episodes of traumatic GHJ luxation^(1-5)^; however, like in the present case, the history of luxation is not always well established^(3)^. As our patient was initially assisted in other service, such a possibility cannot be completely ruled out.

The clinical presentation of glenohumeral interposition of rotator cuff stumps also includes pain and varied degrees of functional limitation or joint blockage. The clinical diagnosis is difficult to be made, and suspicious should be raised in cases where previous radiographic images and those obtained after articular reduction attempts demonstrate persistence of subluxation or articular space widening^(2)^. The radiological signs are subtle, but should be taken into consideration in the clinical context.

In the present case, as well as in the literature review, the authors highlight the role played by MRI in the identification of post-trauma interposition of soft tissues in the GHJ^(1,2,5)^. Such a role is not restricted to cases involving the shoulders, and MRI has been utilized, for example, to detect post-trauma periosteal interposition in growth cartilage fracture in children and adolescents^(11,12)^. In case of irreducible GHJ luxation, computed tomography may be utilized to better identify bone fragments blocking the reduction, but such a method is limited to evaluate soft tissues^(2-5)^.

The management of traumatic rotator cuff injury with tendons entrapment in the GHJ should be surgical, and an early diagnosis can minimize the damages to the involved muscle bellies and tendons, improving the postoperative results^(2,5)^.

Because of its rarity, such a condition may be easily neglected at emergency settings^(1-3)^, and a previous knowledge about this entity by radiologists and orthopedists is critical for a correct diagnosis and institution of an appropriate treatment.

In the present case, the authors conclude that the evaluation by MRI was appropriate to identify the post-trauma rotator cuff tendons entrapment in the GHJ.
